# COVID-19 and Vitamin D Misinformation on YouTube: Content Analysis

**DOI:** 10.2196/32452

**Published:** 2022-03-14

**Authors:** Emma K Quinn, Shelby Fenton, Chelsea A Ford-Sahibzada, Andrew Harper, Alessandro R Marcon, Timothy Caulfield, Sajjad S Fazel, Cheryl E Peters

**Affiliations:** 1 Department of Occupational and Environmental Hygiene School of Population and Public Health University of British Columbia Vancouver, BC Canada; 2 CARcinogen EXposure Canada Faculty of Health Sciences Simon Fraser University Vancouver, BC Canada; 3 Cancer Epidemiology and Prevention Research Department Cancer Care Alberta Alberta Health Services Calgary, AB Canada; 4 Department of Community Health Sciences Cumming School of Medicine University of Calgary Calgary, AB Canada; 5 Health Law Institute University of Alberta Edmonton, AB Canada; 6 Faculty of Law University of Alberta Edmonton, AB Canada; 7 Department of Oncology Cumming School of Medicine University of Calgary Calgary, AB Canada

**Keywords:** COVID-19, vitamin D, misinformation, YouTube, content analysis, social media, video, infodemic, risk, prevention, health information, immunity, immune system, supplements, natural medicine

## Abstract

**Background:**

The “infodemic” accompanying the SARS-CoV-2 virus pandemic has the potential to increase avoidable spread as well as engagement in risky health behaviors. Although social media platforms, such as YouTube, can be an inexpensive and effective method of sharing accurate health information, inaccurate and misleading information shared on YouTube can be dangerous for viewers. The confusing nature of data and claims surrounding the benefits of vitamin D, particularly in the prevention or cure of COVID-19, influences both viewers and the general “immune boosting” commercial interest.

**Objective:**

The aim of this study was to ascertain how information on vitamin D and COVID-19 was presented on YouTube in 2020.

**Methods:**

YouTube video results for the search terms “COVID,” “coronavirus,” and “vitamin D” were collected and analyzed for content themes and deemed useful or misleading based on the accuracy or inaccuracy of the content. Qualitative content analysis and simple statistical analysis were used to determine the prevalence and frequency of concerning content, such as confusing correlation with causation regarding vitamin D benefits.

**Results:**

In total, 77 videos with a combined 10,225,763 views (at the time of data collection) were included in the analysis, with over three-quarters of them containing misleading content about COVID-19 and vitamin D. In addition, 45 (58%) of the 77 videos confused the relationship between vitamin D and COVID-19, with 46 (85%) of 54 videos stating that vitamin D has preventative or curative abilities. The major contributors to these videos were medical professionals with YouTube accounts. Vitamin D recommendations that do not align with the current literature were frequently suggested, including taking supplementation higher than the recommended safe dosage or seeking intentional solar UV radiation exposure.

**Conclusions:**

The spread of misinformation is particularly alarming when spread by medical professionals, and existing data suggesting vitamin D has immune-boosting abilities can add to viewer confusion or mistrust in health information. Further, the suggestions made in the videos may increase the risks of other poor health outcomes, such as skin cancer from solar UV radiation.

## Introduction

The SARS-CoV-2 virus outbreak is a serious global threat, accompanied by an “infodemic” of health misinformation and disinformation [1]. The difference between misinformation and disinformation is based on the intent of the creator or sharer; misinformation is false but not intended to cause harm, while disinformation is deliberately created or shared to mislead or manipulate its audience [2]. Both can be damaging to public health and trust. Although social media can be a valuable tool to share health messaging for free, where it is widely available worldwide [3,4], the overabundance of both accurate and inaccurate health information available to the general public through mainstream and social media can lead to risky health behaviors and, in some cases, even death [5]. There are many factors that influence the consumption of online health misinformation. For example, a recent work by Scherer et al [6] showed that people who are susceptible to misinformation on 1 topic are more likely to be influenced by a variety of misinformation and that those with less education and health literacy, less trust in the health care system, and more positive views toward alternative medicine are also more susceptible to belief in misinformation.

Research has shown that people go online to investigate and diagnose symptoms, to look up treatments and alternative treatments, to research information provided by health care professionals, to research personal as well as public health concerns and topics, to engage with others who have similar health conditions or concerns, and to research and rank health care providers [7,8]. People who use social media for health information face increased exposure to misinformation [9], which in turn can influence their health-related decisions [10]. The explanations of why some are more susceptible to health misinformation are complex, yet research shows that political ideology [11], media use, and trust in government, science, and health authorities can all play influential roles [12].

YouTube is a video-sharing platform visited by approximately 2 billion viewers daily [13]. Over 70% of the videos viewed on YouTube.com are accessed via mobile devices, suggesting that information and entertainment available on the platform are easily accessible in a variety of environments, and YouTube.com is 1 of the most accessed websites [14,15]. In a survey conducted by the Health Information National Trend Survey (HINTS), 8 of 10 people seek health information on the internet [14,16]. Evidence suggests that people use social media to access health information because it can supplement information provided by their health care providers and provide social supports [17].

Despite being a potentially positive source of health information for many, misinformation and disinformation are prevalent on YouTube [15,18]. Currently, YouTube has practices in place to prevent the spread of harmful misinformation [19], though clearly not enough [20]. Health information may be presented in a way that makes it challenging to differentiate the accurate from the inaccurate or to identify misleading statements [21]. Some health professionals take part in spreading misleading opinions and misinformation, adding to the difficulty viewers can experience navigating accurate versus inaccurate health information online [22].

SARS-CoV-2 and the disease it causes (COVID-19) have had an impact on day-to-day life, employment, health care, and general sanitation practices [23]. By April 4, 2020, more than 1 million cases of COVID-19 were confirmed worldwide [24]. At 2 years into the pandemic (as of December 13, 2021), cases had risen to 270 million, accounting for over 5 million deaths. The World Health Organization has provided recommendations for staying healthy and preventing the spread of the virus [25]. Several vaccines are now available in many countries, and efforts to vaccinate large proportions of the population are of paramount importance to curbing the spread of COVID-19, but as of the date of this publication, there is no known cure for COVID-19 [26]. Despite this, there has been an influx of social media posts claiming that an array of substances have preventative or curative properties against COVID-19 and selling dubious “immune boosting” kits, home test kits, and personal protective equipment [27]. Examples of the fake prevention and treatment products promoted on Twitter and Instagram include a mix of so-called immune-boosting supplements (eg, essential oils, some foods, colloidal silver) and unproven pharmaceutical treatments (eg, hydroxychloroquine, remdesivir) [27].

**Figure 1:**
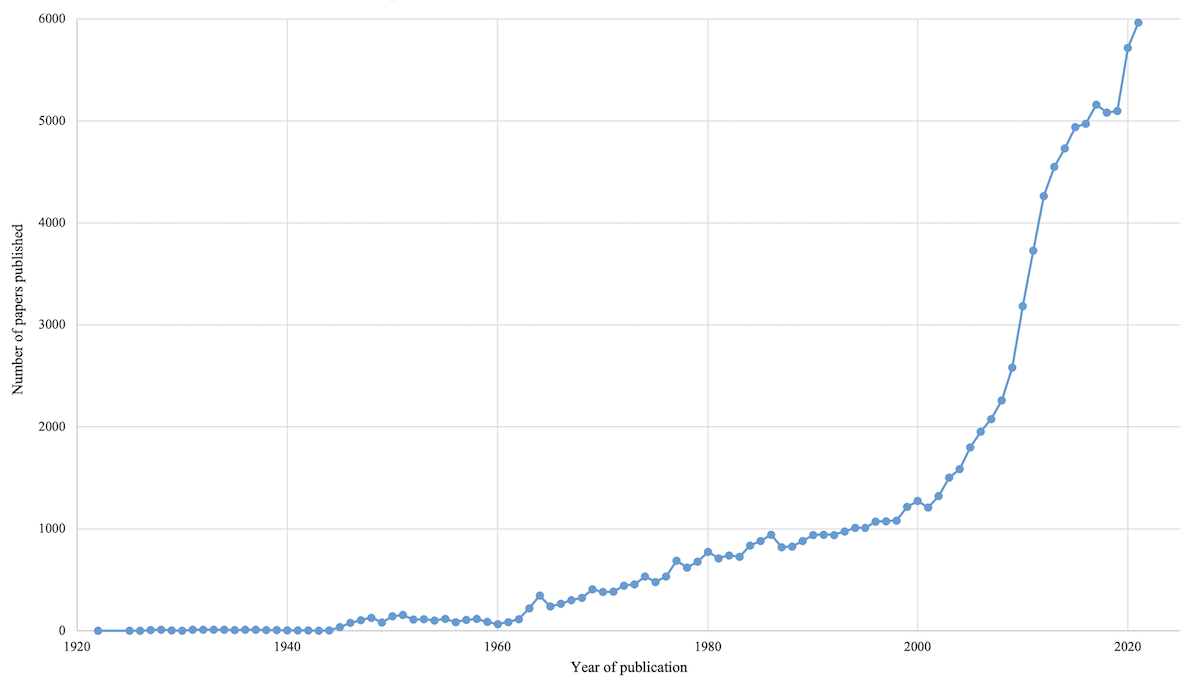
Number of publications over time in PubMed for 'vitamin D', 1922-2021.

One frequently amplified dietary supplement (during the pandemic but certainly not a new trend) is vitamin D, available through consumption of naturally occurring or fortified foods, supplementation, and synthesized naturally in the skin after UV radiation exposure [28]. The current understanding of the important functions of vitamin D in the body include regulation of serum calcium and phosphate homeostasis, which aids in the maintenance and development of bones. Foods naturally containing vitamin D include fatty fish, fish liver oil, and egg yolks, while other common foods often fortified with synthetic vitamin D include milk, margarine, bread, and orange juice [29]. The fortification is done to prevent vitamin D deficiency, which can lead to rickets. Beginning in the early 2000s, an increasing number of studies investigated vitamin D as a preventive or curative agent for a wide variety of ailments and this has only increased over the past 20 years (Figure 1, data from PubMed), with an evident spike in 2020-2021. Even though it has been extensively studied as a potential preventive agent for a variety of cancers and other chronic and infectious diseases, evidence for the benefits of supplementation have largely failed to show appreciable beneficial effects on human health (besides in cases of extreme deficiency) [29-31]. Early in the COVID-19 pandemic, a correlation between lower vitamin D levels and severity of outcomes was reported across many studies, which led to the idea that supplementation (either preinfection as a preventive agent or postinfection to support treatment) may play a role in pandemic control [32,33]. The most recent meta-analysis on the topic concluded that vitamin D deficiency can increase the susceptibility to severe COVID-19, but noted that the included studies suffered from high risk of bias and significant heterogeneity and that several of the randomized control trials were too widely heterogenous to include in meta-analysis [34].

Recommended daily supplementation doses of vitamin D range from 400 IU to 800 IU, depending on the age and condition of the individual, and consuming excess of 4000 IU is generally not recommended as safe [28]. Several companies have advertised supplements (including ones that contain vitamin D) as having immune-boosting properties and thus are potentially profiting from the misinformation infodemic accompanying the COVID-19 pandemic [35]. Recommendations to take a supplement without adequate medical reference or advice may be harmful to the individual and can lead to hypercalcemia and even death in rare cases [36].

The aim of this study was to qualitatively analyze how vitamin D was presented in association with COVID-19 in YouTube videos shared in 2020. Inaccurate or inappropriate messaging regarding vitamin D and COVID-19 may be problematic for a host of reasons, including causing people to take supplements to feel that they are safe from a highly infectious disease that requires vigilant public health behaviors and vaccination. In addition, it may help to drive and legitimize scientifically inaccurate conceptions of immune boosting.

## Methods

### Data Collection

We searched YouTube.com for the keywords “COVID,” “coronavirus,” and “vitamin D” on June 10, 2020, and again on December 7, 2020. We used the Google Chrome web browser, and to limit bias associated with a personal Google.ca account or prior search history, an incognito web browser was used, and no Google account was linked to the search. Browser history was also erased, including cookies and cache, prior to conducting each search. Default search filters were not modified to present the findings in the most common search order, in order of relevance, as it would appear when a person usually searches for a video on YouTube. During each data collection event, search results were collected from the first 3 pages of results, or 60 videos, as previous studies have suggested that most individuals do not view results past the third page [37]. URLs from the first 60 posts of search results were transferred to Microsoft Excel, along with descriptive characteristics, such as the result number, post title, account name, date posted, engagement (thumbs up, thumbs down, and number of comments) on the date of data collection, and type of account.

Only English YouTube videos that discussed COVID-19 *and* vitamin D were included. Videos were excluded if they discussed only 1 of the 2 information categories (ie, COVID-19 or vitamin D). Duplicate videos were also removed.

### Content Analysis

Content analysis is a method of taking valid and replicable inferences from a group of texts for the purpose of specific research context, as used in previous studies [38]. The posts were analyzed using a coding framework (Multimedia Appendix 1) similar to previous social media content analysis studies conducted by our team [39-41] and a codebook developed a priori that was based on COVID-19 themes seen in previous studies and vitamin D–specific themes. Audio and visual content was coded together to ensure the unique impact of YouTube videos was coded appropriately. During each data collection event, a team of 2 coders (authors SF/EQ, SLF/CFS) used the code book to code all videos for useful (all accurate information) or misleading (any inaccurate or misleading content) COVID-19 and vitamin D information, unsafe sun exposure recommendations, and confusing correlation with causation. In particular, videos were tagged as misleading if they included information that vitamin D prevents or cures COVID-19, which is a statement that is not in line with the current evidence base, which presently only concludes a correlation between the 2 [34]. The video content was then recorded for areas of interest described in the codebook: a set of codes and inclusion criteria/descriptions developed a priori. If differences in coding results could not be resolved through a consensus-driven discussion between coders, the senior author (CP) was used as a third reviewer to reach consensus. During analysis, the account holder was investigated to determine the type of user (medical professionals included users who stated on their account that they are qualified and work in a medical field; this excluded chiropractors and naturopaths).

### Statistical Analysis

In addition to qualitative content analysis, we calculated simple descriptive statistics to investigate the prevalence of misinformation in our collected videos, as well as whether engagement metrics differed by video accuracy. Bivariate analyses were conducted using various video metrics and parameters to assess potential associations and patterns in the collected data. These analyses consist of generating 2-way tables that describe the relationship between multiple pairs of individual metrics. Chi-square or Fisher exact tests were conducted depending on the appropriateness of the cell size to assess associations between metrics, where strengths of association are represented using *P* values [42].

## Results

### Data Collection

In total, 77 (64.2%) of 120 YouTube videos screened were included in our analysis. We excluded 27 (22.5%) YouTube videos as they did not present information on vitamin D *and* COVID-19 (ie, the videos only discussed 1 of the 2 topics of interest). Videos were also excluded due to duplication (n=13, 10.8%), non-English language (n=2, 1.7%), or blocking by YouTube on copyright grounds (n=1, 0.8%).

The 77 videos included in our study had a total of 10,225,763 views at the time of our analysis. Videos posted by medical professionals accounted for the majority of the videos (n=34, 44%) included in our analysis, followed by “other,” for example, personal (n=24, 31%), and news (n=19, 25%) account types.

### Accuracy and Engagement Metrics

**Figure 2:**
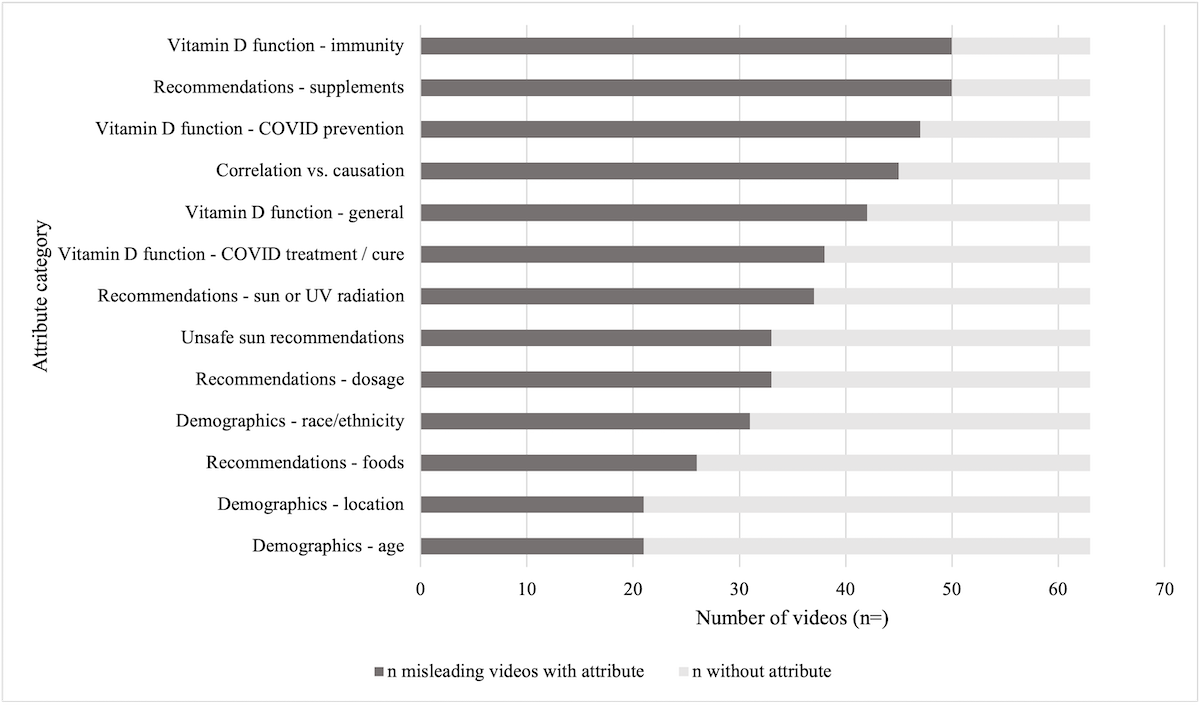
Attributes and frequency of appearance in videos coded as misleading (n=62).

Nearly three-quarters (57/77, 74%) of the videos contained at least some misleading information about COVID-19, and 60 (78%) contained misleading information about vitamin D (Table 1, Figure 2). Indeed, most videos (55, 71%) contained at least some misleading information about *both* COVID-19 and vitamin D, and only 15 (19%) videos were accurate in their statements about vitamin D and COVID-19. A minority of videos provided a mix of useful and misleading information across the 2 topics (Table 1). For further analysis on accuracy, we classified the videos as misleading if they had misleading information on vitamin D, COVID-19, or both (ie, 7 [9%] videos with some useful and some misleading information were labeled as misleading overall).

When examining accuracy by account type, we found that most of the useful videos were shared by medical professional accounts (12/15, 80%). The remaining few useful videos were shared by either news organizations or the “other” type (Table 2). Interestingly, medical professionals also shared over three-quarters of the misleading videos (22/62, 35%), although the “other” account type shared the most misleading videos (23/62, 37%) of all misleading videos. There was a statistically significant difference (*P*=.01) between the types of accounts sharing misleading versus useful videos, with medical professionals more likely to share useful information, but medical professionals still mostly shared misleading information on COVID-19 or vitamin D (Table 2).

The number of views, comments, and thumbs up/thumbs down are summarized by overall video accuracy in Table 3. A comparison of mean values suggests that YouTube videos containing useful vitamin D information had greater viewer engagement overall, including a greater number of views; however, the video with the single greatest number of views (1,895,430) was misleading about both COVID-19 and vitamin D. These differences, however, were not statistically significant.

**Table 1:** Accuracy of vitamin D information vs accuracy of COVID-19 information (N=77).

Misleading/useful information^a^	Misleading COVID-19 information, n (%)	Useful COVID-19 information, n (%)	Total, n (%)
Misleading vitamin D information	55 (96)	5 (25)	60
Useful vitamin D information	2 (4)	15 (75)	17
Total, n (%)	57 (100)	20 (100)	77 (100)

^a^Fisher exact test: *P*<.001.

**Table 2:** Accuracy of information by account type (N=77).

Account type	Misleading information, n (%)	Useful information, n (%)	Total, n (%)	*P* value
Medical professional	22 (35)	12 (80)	34	.01
News	17 (27)	2 (13)	19	.01
Other	23 (37)	1 (7)	24	.01
Total	62 (100)	15 (100)	77 (100)	.01

**Table 3:** Overall accuracy by engagement metric (N=77).

Engagement metric^a^	Misleading information				Useful information				*P* value
	n	Mean (SD)	Range	n		Mean (SD)	Range		
Views	62	108,436 (278,604)	8-1,895,430	15		242,846 (470,504)	62-1,786, 066	.21	
Comments	62	603 (1397)	0-8711	15		1702 (2992)	2-10,760	.15	
Thumbs up	62	2639 (6212)	0-40,000	15		6153 (11,774)	0-48,000	.24	
Thumbs down	62	64 (154)	0-1000	15		272 (710)	0-2800	.08	

^a^At the time of data collection.

### Attributes and Themes

**Figure 3:**
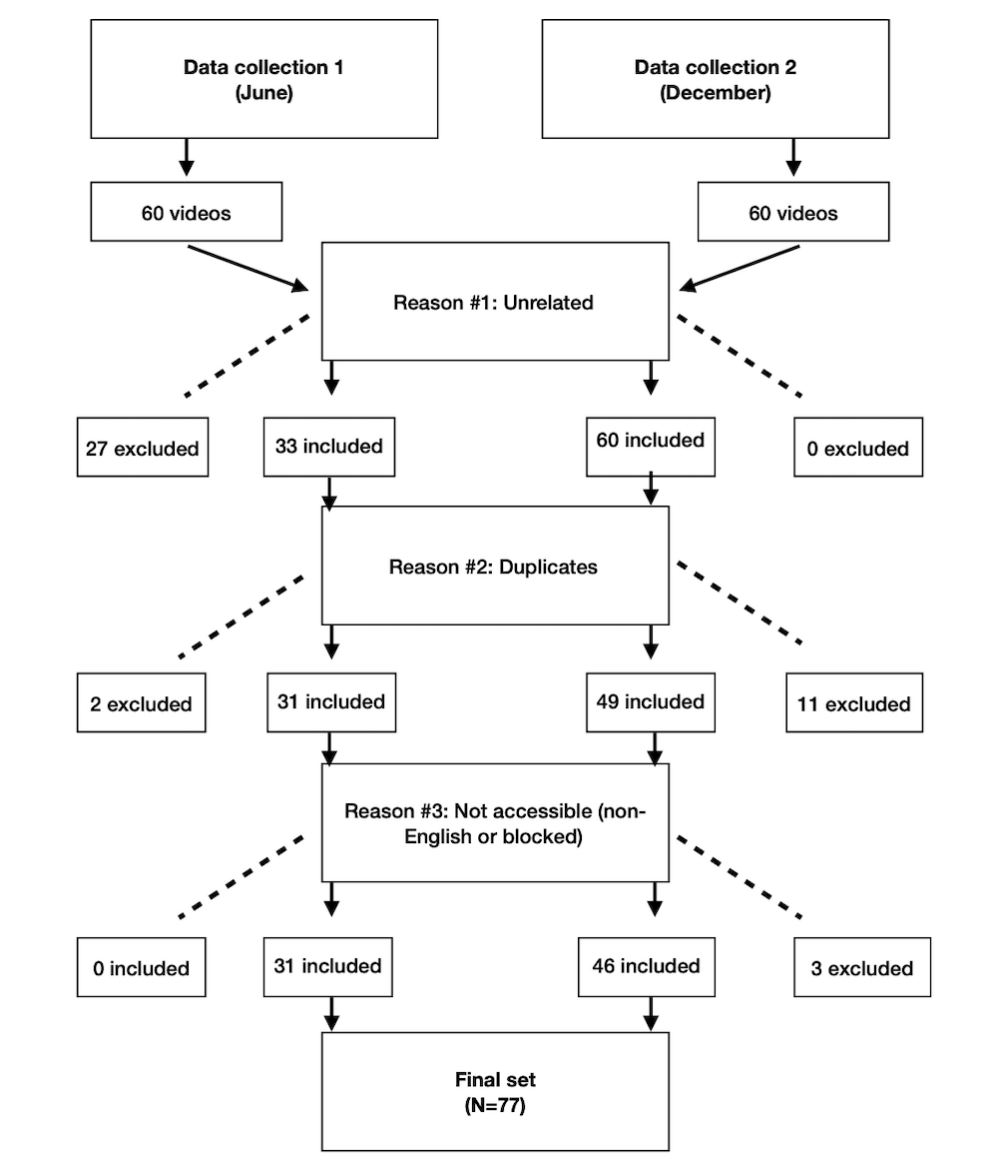
Data inclusion and exclusion criteria.

**Figure 4:**
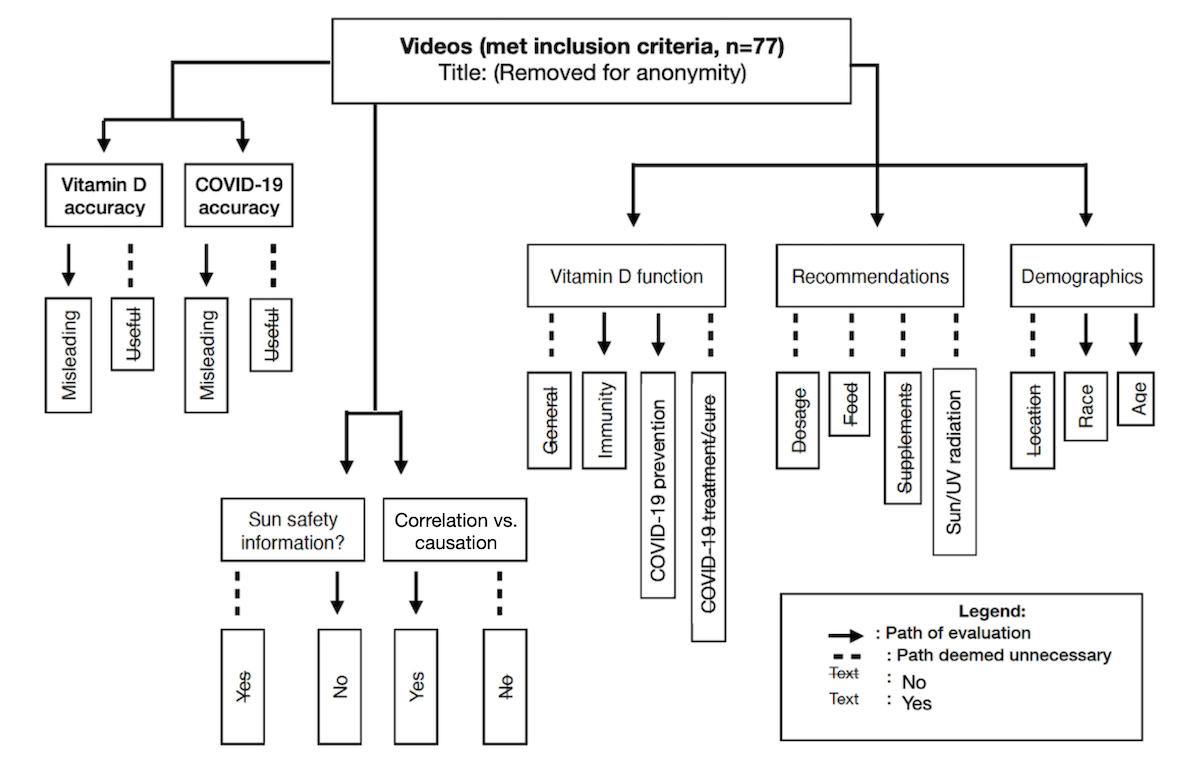
Sample of coding system.

The number of videos per attribute category demonstrated the overall themes shared by the content creators (Figure 3), as well as a sample coding system (Figure 4). Approximately half (37/77, 48%) of the videos recommended that people engage in unsafe sun (does not fit within recommendations [43]) or UV-related behaviors in an effort to improve their vitamin D status (eg, “It’s free; just go out in the sun”). Intentional (unprotected) sun exposure was recommended in videos, including the idea to “seek direct sun exposure for 20-60 minutes with minimal clothing,” as well as the suggestion that those who have higher levels of melanin in their skin increase their sun exposure, even extremes such as “Stand naked in direct sunlight for a minimum of 20 minutes” or “Never use sunblock.” Sunlight was occasionally presented as the “only” or “best” source of vitamin D, recommending “exposure during peak UV hours for optimal absorption.” Such information was coded as misleading due to the contrasting statements made by sun safety organizations and existing literature recommendations, such as (but not limited to) avoiding direct unprotected sun exposure of over 15 minutes, avoiding exposure during peak UV hours, and wearing (and reapplying) sunscreen and protective clothing when sun exposure is unavoidable [43]. Many videos did, however, provide recommendations on how to safely generate vitamin D from the sun (eg, minimizing exposure with the use of sunscreen, clothing, or seeking shade).

Of additional concern, a total of 45 (58%) of 77 videos confused correlation with causation either directly or implicitly. Relevant statements included suggestions that the global COVID-19 pandemic is actually a “pandemic of insufficient vitamin D levels.” Concerning statements were also made regarding the state of the scientific evidence between vitamin D levels and COVID-19, suggesting that the “evidence is now so strong” and “overwhelming.” Videos also suggested that “every public health official should be recommending it [vitamin D for COVID-19],” and doing so “could save the lives of millions.” The notion that “there is no harm in adding a vitamin D supplement to your daily routine” was found in several videos, despite evidence in the literature that demonstrates an overdose of vitamin D can be harmful [36]. Overall, the suggestion of vitamin D supplements being a safe and easy way to “boost immunity” was a common thread in many videos.

Videos commonly discussed the general function of vitamin D (55/77, 71%) and how vitamin D functions in overall immunity (62/77, 80%). Many videos highlighted that vitamin D is known as the “sunshine vitamin” and stated that a considerable proportion of the global population is vitamin D insufficient or deficient. Some videos also included in-depth scientifically supported details regarding vitamin D production, metabolism, and associated mechanisms of action.

Most of the videos (54/77, 70%) explicitly discussed vitamin D as a COVID-19 primary prevention method or to prevent more severe outcomes. Although not all these videos provided misleading information on these topics, the majority (46/54, 85%) of videos including these topics did directly state or imply that vitamin D can prevent or cure COVID-19, which is not supported by current scientific evidence and is thus misleading. Further, 41 (53%) of the 77 videos included comments on the ability of vitamin D to treat or cure COVID-19, and 37 (90%) of these contained misleading information on this topic. Examples of the types of misleading messages included that vitamin D is an “effective treatment” for COVID-19 and could be “lifesaving” and suggested that physicians should provide it to patients infected with COVID-19.

The 8 (10%) of the 77 videos that contained useful information about the potential for vitamin D to prevent COVID-19 or reduce severe disease generally informed viewers that ongoing studies were investigating the theory of vitamin D preventing COVID-19 and outlined proposed mechanisms of action. Useful videos also noted that the current state of the science “does not prove” that vitamin D deficiency increases susceptibility to COVID-19 infection. In comparison, misleading videos encouraged individuals to increase their vitamin D intake to “reduce their likelihood of catching it (COVID-19)” because there is a “strong relationship” between vitamin D status and COVID-19 infection rates. The 4 (10%) of 41 videos that contained useful information about the potential for vitamin D to help treat or cure individuals included similar messages as the useful videos about vitamin D being a potential preventative agent against COVID-19. Useful videos discussed how some hospital-based pilot studies are including vitamin D (or calcifediol) as part of experimental treatment protocols for COVID-19 patients.

Generally, there was a mix of useful information (eg, recommendations to “discuss supplementation and dosage with your physician”) concerning vitamin D recommendations, particularly regarding supplementation (eg, “immune boosting” to preventing COVID-19) and “prescription level” dosage (eg, up to 60,000 IU a day). Several videos stated that “vitamin D supplements amplify immune function” or provide a “boost” to the body in fighting off the virus. A common supporting theory to the claims of vitamin D having a protective factor was that supplement use will “reduce inflammation in the body.” Several videos did suggest consulting with a physician prior to taking supplements, while others suggested starting at a base dosage of 2500 IU daily. We also analyzed the videos for the theme of vitamin D recommendations related to the subthemes of vitamin D dosage, supplements, food sources, and sun or UV radiation exposure. Many of the videos did provide a recommendation (or recommendations) to viewers, with vitamin D supplements (59/77, 77%) being the most common, followed by sun or UV radiation exposure (42/77, 55%), vitamin D dosage (41/77, 53%), and food sources (31/77, 40%).

The videos also discussed the theme of demographics and risk, including aspects of ethnicity, location, and age in relation to vitamin D and COVID-19. Ethnicity was discussed in approximately half of the videos. This content typically focused on how darker-skinned individuals may be more susceptible to vitamin D deficiency and this could support the understanding of racial (and ethnic) differences in severe COVID-19 outcomes. Videos that discussed location in relation to vitamin D and COVID-19 (28/77, 36%) commonly described the increased risk of vitamin D deficiency in northern latitudes or a hypothesized COVID-19 “latitude gradient.” Lastly, videos that had a theme of age (also 28 [36%] videos) generally described how older individuals (ie, greater than 60 years old) may be more susceptible to vitamin D deficiency and, therefore, COVID-19.

## Discussion

### Principal Findings

Our results provide evidence that videos available on YouTube contribute to the infodemic, which may lead to misunderstanding and confusion among viewers. Overall, the results of our study indicated that the majority of videos contain misleading information about both COVID-19 *and* vitamin D, frequently implying in a causal manner that vitamin D supplementation reduces COVID-19 incidence. This type of misinformation is particularly concerning from a public health perspective, given the audience and its susceptibility to be influenced by health information [44]. Although some videos were careful to explicitly state the difference between correlation and causation, others went on to state a direct association between vitamin D and COVID-19, despite the lack of reliable data [30].

Misleading videos generally overstated our current understanding of the relationship between vitamin D and COVID-19 or presented a 1-sided view of the current research (ie, strictly sharing research in support of an association between vitamin D status and COVID-19 outcomes). In addition to sharing selective and misleading messages, the available information was frequently confusing by stating that vitamin D has preventative or has curative abilities against the COVID-19 virus. Of great concern, misleading videos also suggested or directly stated there was no evidence to support COVID-19 public health prevention measures (eg, masks, social distancing, lockdowns) despite the mounting evidence supporting decreased transmission rates with the preventative measures [25]. The most recent meta-analysis on vitamin D as a preventive or curative treatment for COVID-19 did report correlations between levels of vitamin D and COVID-19 outcomes, but the authors were careful to note that the available studies had a high risk of bias and heterogeneity [34]. One bias of particular concern was related to the timing of vitamin D ascertainment, which in many studies was done at the time of diagnosis or hospital admission, which obscures the ability to determine causation (as compared to correlation). A further complication is that we know that circulating vitamin D concentration decreases in times of acute illness or inflammation [45]. This means that given the types of studies available, it is impossible to ascertain whether having higher vitamin D prevents COVID-19 (or severe outcomes), whether COVID-19 inflammation causes lower vitamin D levels, or that vitamin D is a marker of the underlying health status—or indeed some combination of all 3 scenarios.

Vitamin D supplementation recommendations were made in many of the videos that inappropriately associated vitamin D supplementation with reduced risk of contracting COVID-19, often suggesting a dosage higher than standard guidelines [28] or not recommending inquiring about vitamin D recommendations from a family physician (such as based on a confirmed, clinically relevant deficiency). Dietary sources of vitamin D were discussed; however, they were often deemed less valuable than a supplement or solar UV source. Encouraging members of the public to purchase supplements or engage in risky health behaviors for unproven benefits is concerning to public health researchers, tying together health risks and poor health outcomes, such as skin cancer, with the COVID-19 pandemic [46,47]. Several misleading videos also suggested that all individuals should take a vitamin D supplement as they are without risk, readily available, and cheap, or even suggested the use of an extremely high-dose [28] or “prescription level” vitamin D regimen (eg, 60,000 IU/day) to prevent COVID-19 illness and to “boost the immune system.” These videos also commonly described the global population as being vitamin D deficient/insufficient and claim that this is the “real root cause” of the pandemic.

Unsafe sun exposure was a common recommendation in order to increase vitamin D levels, with claims that intentional sun exposure was the “best” option for increasing immunity. Recommending unsafe exposure to UV radiation is alarming, particularly when it is classified by the International Agency for Research on Cancer (IARC) as a known skin carcinogen [48] with other well-documented negative health effects [49]. Although sunlight is a known source of vitamin D [50], studies have shown that the DNA damage and elevated skin cancer risk associated with direct sun exposure outweigh the vitamin D status, particularly when replaceable by diet or supplements [51].

Medical professionals have a highly influential position on online platforms due to the assumption they are sharing accurate and reliable information learned through their professional education [52]. Although COVID-19 is a relatively new area of research, it is not encouraging to observe accredited medical professionals sharing potentially dangerous health misinformation, including suggesting individuals overdose on a vitamin or seek intentional risky sun exposure, in turn increasing their risk of skin cancer or other poor health outcomes [20,53,54]. It would be advisable for medical professionals making informational videos on YouTube that they use their platform to share only reliable and accurate information [52], rather than speculative claims for holistic measures (particularly when for personal financial gain), as it has been demonstrated that consumers of social media place more trust in these professionals [52]. Although financial gain (eg, from supplement sales) is 1 reason that some health professionals may share misinformation, this is unlikely to cover all situations. Indeed, physicians and other health care professionals can be susceptible to believing ideas that are at least biologically plausible or where they have trusted colleagues who share in the belief [55]. Additionally, these professionals have a strong desire to alleviate suffering and could have a lower threshold for what constitutes “evidence” in the prevention or treatment of a novel virus [56,57]. It is assumed or expected by many viewers that a medical professional would only share reliable and accurate information [52], although from our results, it is clear that this is not always the case. This could alter the public’s sense of medical knowledge and potentially lead to doubt in the health system.

Not all information within the YouTube videos analyzed included misinformation; some of the videos were useful and could provide viewers with valuable information pertaining to their health. Overall, we found useful information was also shared, including guidance on the potential benefits and risks associated with vitamin D intake and the current epidemiology of COVID-19. Other useful videos shared several studies that both supported *and* refuted an association between vitamin D and COVID-19. The videos containing useful information were also found to describe the state of current science, the limitations of current research studies, and the need for additional research before making any supplement or other recommendations. Social media can be a valuable and inexpensive method of sharing health information widely with the public, as long as it is clear and accurate [46].

Despite some of the videos containing useful information, the overall recommendation of supplements contributes to the concerning theories of “immune boosting” holistic approaches to health. This is a dangerous place that lacks sufficient scientific evidence to support the claims [53,54]. The pandemic has lead wellness influencers and companies promoting “immune boosting” products to capitalize on the vulnerability of the unprecedented times of the pandemic. Commercial interest in the “immune boosting” products, as noted in the study by Wagner et al [47], was present with most “immune boosting” posts on Instagram. Similarly, among general Google searches, evidence-based claims were paired with “immune boosting” theories, inadvertently legitimizing the concepts [35]. In the case of medical professionals with YouTube accounts, their position of authority may be inadvertently legitimizing the claims stated about vitamin D *and* COVID-19, simply because viewers assume a medical professional would only share accurate reliable information [52].

### Limitations

This study had some important limitations that should be mentioned. First, we designed our study to collect 60 videos at 2 separate time points, for a total of 120 videos, but many of the videos captured by our search strategy only included information on 1 of our topics of interest (eg, either COVID-19 or vitamin D but not both). This created a smaller data set in our sample than we anticipated. However, 1 of the main drivers of our inquiry was how members of the general population interface with YouTube and what videos they would be likely to see based on simple searches, not to find every video possible using more complex Boolean strings. Therefore, we are confident that the sample of videos we collected was representative of real-world information that is easily accessible to an average user. We also had 2 separate coding teams for the 2 time points of our study, which could have introduced differences in coding across the time points. However, all coders were central members of an experienced team working on similar topics and from the same codebook, led by the same senior scientist, who also carefully reviewed all videos and coding to ensure consistent approaches across the phases.

### Conclusion

In conclusion, the results of our study suggest that confusing messaging about vitamin D as having preventative or curative abilities against/for COVID-19 is prevalent on social media and is dominating the online narrative. Concerns surrounding the type of individuals spreading this type of health misinformation are unique in the unprecedented times of a global pandemic, where the public may be anxiously seeking advice about how to remain healthy [3,58]. Easily accessible online platforms hold the potential to decrease the spread of SARS-CoV-2; however, if misinformation is shared publicly, it can lead to increased viral spread or the increased presence of other poor health outcomes either immediately or in the future (such as skin cancer from intentional UV radiation exposures) [59]. This study is an important contribution for public health, as it demonstrated that health professionals are a significant source of misleading information on the relationship between vitamin D and COVID-19 infection and severity. The practical next steps to address this challenge include the sharing of antimisinformation efforts as well as prebunking or debunking methods to curb risky “immune boosting” behaviors on social media in order to deter the avoidable negative health consequences of unnecessary supplementation [60].

## Appendix

Multimedia Appendix 1 Attributes and attribute options, and themes and subthemes.
