# Supplementary material for: COVID-19 and Vitamin D Misinformation on YouTube: Content Analysis
Source: JMIR Infodemiology. 2022 Mar 14;2(1):e32452. doi: 10.2196/32452 (PMC8924908; doi:10.2196/32452)
Supplement: Multimedia Appendix 1 [file infodemiology_v2i1e32452_app1.docx]

## Supplementary material:

## Table 1: Attributes and attribute options

| Attribute | Sub-category (attribute drop down options) | Definition |
| --- | --- | --- |
| Accuracy | Vitamin D - Useful | Information about Vitamin D that is consistent with existing scientific literature and is presented in a way that is appropriately weighted to describe action that individuals should take regarding vitamin D consumption. |
|  | Vitamin D - Misleading | Information about vitamin D that is contradictory to existing literature or overstating the effects and/or application suggested in literature. |
|  | COVID-19 - Useful | Information about COVID-19 that is consistent with existing literature findings, symptoms and rates, and is presented in a way that is appropriately weighted to describe action that individuals should take regarding COVID-19 risk. |
|  | COVID-19 - Misleading | Information about COVID-19 that is contradictory to existing literature or overstating the risk and/or prevention methods suggested in literature. |
| Unsafe sun or UV exposure recommendation | Yes/No | Whether or not the post suggests unsafe sun exposure as a method of achieving adequate vitamin D. |
| Correlation vs. causation confusion | Yes/No | Whether or not the post contains information that implies or explicitly states vitamin D correlation with COVID-19 infection or outcomes. |

Table 2. Themes and sub-themes

| Theme | Sub-Theme | Definition |
| --- | --- | --- |
| Vitamin D function | General | General purpose and function of vitamin D in the human body |
|  | Immunity | Suggestion of how vitamin D influences immunity |
|  | COVID-19 prevention | Suggestion of how vitamin D can be used to prevent COVID-19 |
|  | COVID-19 treatment or cure | Suggestion of using vitamin D to treat and cure COVID-19 |
| Recommendations | Dose | Recommendations of vitamin D dosage |
|  | Foods | Foods that contain high amounts of vitamin D and are recommended for consumption when seeking vitamin D in the diet |
|  | Supplements | Supplements recommended to increase vitamin D in the body |
|  | Sun | Recommendations of sun exposure to increase vitamin D in the body |
| Demographics and risk | Location | Focus on individuals at specific locations or latitude |
|  | Race | Suggestions based on racial implications regarding vitamin D and/or COVID-19 |
|  | Age | Suggestions based on age regarding vitamin D and/or COVID-19 |
